# Pyoderma gangrenosum following treatment with azacitidine in a patient with myelodysplastic syndrome

**DOI:** 10.1016/j.jdcr.2026.04.012

**Published:** 2026-04-17

**Authors:** Vandana Dubakula, Callie Fort, Christie Pham, Meaghan Dubin

**Affiliations:** a Baylor Scott & White Health, Round Rock, Texas; b Houston Methodist Hospital, Houston, Texas; c Kelsey-Seybold Clinic, St. Luke’s Medical Tower, Houston, Texas

**Keywords:** azacitidine, myelodysplastic syndrome, pyoderma gangrenosum, painful ulceration

## Introduction

Pyoderma gangrenosum (PG) is an uncommon dermatological diagnosis with reported incidence of 3 to 10 cases/million people/year.^1^^,^^2^ It poses a diagnostic challenge given its rarity and similarity in presentation to more common skin conditions, leading to misdiagnosis and delay in care. Untreated PG can lead to progressive and painful ulceration, secondary bacterial infections, or permanent scarring with functional impairment. Severe cases, especially with uncontrolled disease or immunocompromised patients, have been associated with mortality rates up to 30%.^3^ Clinical clues include a red violaceous wound border, rapidly progressive disease, severe tenderness, and localization of the lesion at the trauma site. Suggestive features should prompt punch biopsy, which can help provide a definitive diagnosis. Immunosuppressive therapy has shown to reduce the size of the ulcer within 1 month. PG is usually associated with autoimmune diseases, such as rheumatoid arthritis and inflammatory bowel disease. Azacitidine-associated PG is exceedingly rare with only 3 reported cases in the literature.^4^, ^5^, ^6^

## Case report

**Fig. 1:**
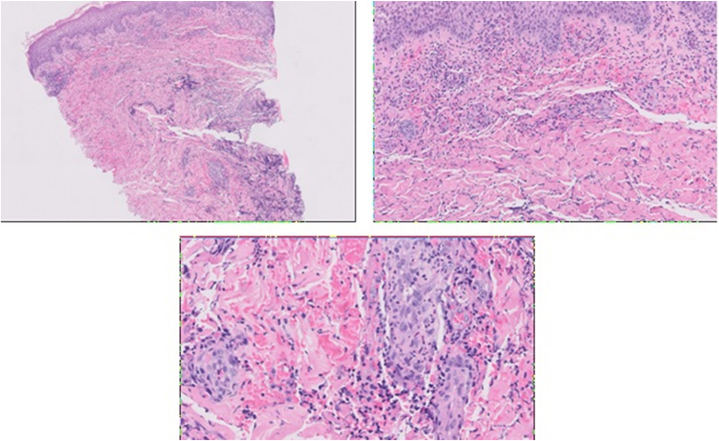
Punch biopsy of the lesion with hematoxylin and eosin staining. Pathology showing superficial and deep perivascular infiltrate composed of lymphocytes and neutrophils at 40× magnification (*top left*), 100× magnification (*top right*), and 200× magnification (*bottom middle*). These findings support a diagnosis of pyoderma gangrenosum.

A 70-year-old woman presented with a new-onset rapidly progressive skin lesion. Her medical history included hypertension, diabetes, history of stage I diffuse large B-cell lymphoma, and treatment-induced high-risk myelodysplastic syndrome (MDS). Four weeks after her first cycle of subcutaneous azacitidine, she developed a painful “nickel-sized” erythematous nodule on the lower portion of the left side of her abdomen (Fig 1, *A*). She was initially prescribed empiric cephalexin for presumed cellulitis. On physical assessment 3 days later, the nodule had progressed to a painful blue-purple ulcer measuring 6 cm × 4 cm with surrounding warm erythema measuring 15 cm × 8 cm (Fig 1, *B*), prompting hospital admission.

**Fig. 2:**
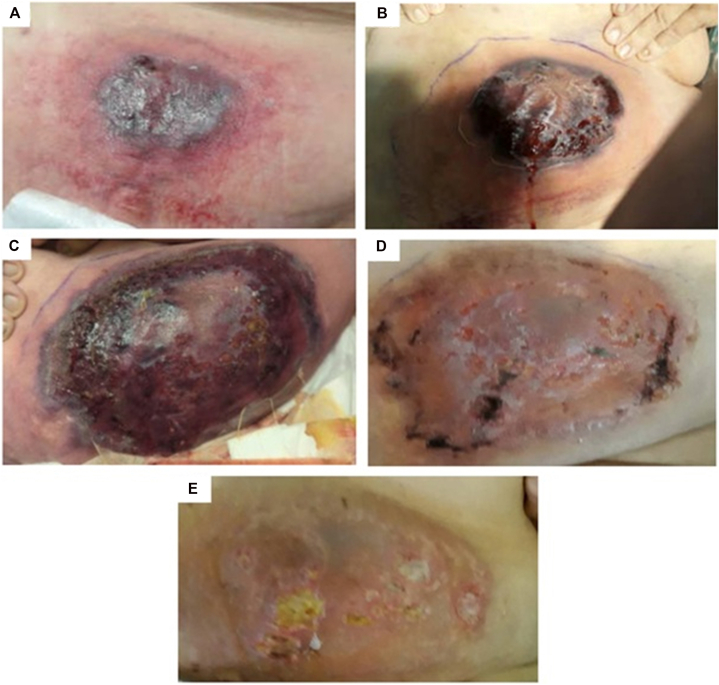
Progression of the pyoderma gangrenosum skin lesion from initial presentation. **(A)** May 16: Initial presentation of the lesion with surrounding erythema. Four weeks prior, the patient received the first dose of subcutaneous azacitidine. **(B)** May 21: rapid progression of the lesion. **(C)** May 23: Further progression of lesion. Biopsy confirmed pyoderma gangrenosum diagnosis at this time. Patient started prednisone and topical tacrolimus. **(D)** June 03: improvement in appearance of the lesion 11 days after starting steroids. **(E)** June 16: appearance of the lesion 24 days after starting steroids.

Given her immunocompromised status, she was started on empiric vancomycin and piperacillin–tazobactam for possible cellulitis. Computed tomography imaging revealed abdominal wall subcutaneous edema and skin thickening without abscess. Punch biopsy cultures grew *Klebsiella pneumoniae*, *Proteus mirabilis*, and *Enterococcus faecalis* and histopathology demonstrated superficial and deep perivascular infiltrate composed of lymphocytes and neutrophils with red blood cell extravasation (Fig 2). The histopathologic differential diagnosis included changes secondary to trauma, medication-induced skin necrosis, infectious process, or PG, the latter especially if continued progression of the skin ulcer. Antibiotics were de-escalated, and the patient was discharged with dermatology follow-up.

Two days after hospital discharge, the ulcer progressed to a 17 cm × 11 cm deeply violaceous plaque with areas of ulceration. There was associated cutaneous erythema, induration, serosanguinous drainage, and tenderness (Fig 1, *C*). The pathological and clinical presentation was deemed consistent with PG. Patient was started on prednisone 60 mg daily and topical 1% tacrolimus, leading to marked improvement within few days (Fig 1, *D* and *E*). The patient was restarted on intravenous azacitidine without further recurrence of PG.

## Discussion

PG is an immune-mediated disorder associated with underlying systemic and/or autoimmune disease. The pathophysiology is not well understood but is most often triggered by trauma (pathergy) and associated cytokine release.^1^ Innate and adaptive immunity are inappropriately activated in the absence of infectious stimulus, resulting in rapidly evolving tissue damage with the initial target thought to be the hair follicle.^7^

The differentials for PG include cellulitis, cutaneous vasculitides, sweet syndrome, cellulitis, and necrotizing fasciitis. Delphi Consensus (2018) outlines diagnostic criteria for PG, requiring major criterion of biopsy demonstrating neutrophilic infiltrate and at least 4 of the following 8 minor criteria: (1) exclusion of infection; (2) pathergy; (3) history of papule, pustule, or vesicle ulcerating within 4 days of appearing; (4) peripheral erythema, undermining border, and tenderness at ulceration site; (5) decreased ulcer size within 1 month of immunosuppressive medications; (6) history of inflammatory bowel disease of inflammatory arthritis; (7) multiple ulcerations, at least 1 on anterior lower leg; and (8) cribriform or “wrinkled paper” scar(s) at healed ulcer sites.^8^

Our patient fulfilled the major criterion and the first 5 of the above 8 minor criteria, shown to yield a sensitivity of 86% and specificity of 90%.^6^ Infection was excluded through negative imaging findings and blood cultures and nonresponse to antibiotics. Recent subcutaneous administration and the rapid progression of the lesion to a painful ulcer with surrounding erythema and induration supported criteria 3 to 4. Prompt improvement after corticosteroid initiation satisfied the final criterion. Although both prednisone and cyclosporine are mainstay immunosuppressants for PG, prednisone was chosen in this case given the patient’s underlying malignancy and other comorbidities. Topical tacrolimus was used as adjunctive therapy alongside systemic corticosteroids to promote local disease control.

Therapy with azacitidine is generally better tolerated and improves overall survival in patients with higher-risk MDS who cannot tolerate intensive chemotherapy or are not candidates for stem cell transplantation. A review of drug-induced PG identified azacytidine (*n =*3) as a possible cause along with isotretinoin (*n =*5), propylthiouracil (*n =*5), and sunitinib (*n =*5). History of cocaine use (*n =*13) was identified as a definite adverse drug reaction.^9^ Although PG is most commonly linked to inflammatory bowel disease and rheumatoid arthritis, it has also been described in association with MDS. The incidence of PG in MDS patients is unknown because of the lack of controlled cohort studies or registry data; however, case reports and series suggest it is a rare complication.^10^

Among 3 reported cases of azacitidine-associated PG, 1 suggested MDS as the trigger, but recurrence after restarting azacitidine implicated this drug as the cause.^6^ Another case involved discontinuing azacitidine and switching to decitabine, without further recurrence of PG.^4^ The third case showed resolution of PG after transitioning to intravenous azacitidine without evidence of recurrence.^5^ Our patient similarly transitioned to intravenous azacitidine without further recorded PG episodes. These cases suggest that PG may arise from overlapping factors, including underlying hematologic disease, local injection site trauma, and medication exposure, and that continuation of azacitidine via an alternative route may be reasonable in selected patients.

This case highlights the importance of rapid recognition of PG and early initiation of immunosuppressive therapy to prevent progression and morbidity. Because azacitidine remains a cornerstone treatment for higher-risk hematologic disease, recognition of PG arising after subcutaneous administration of azacitidine is important, and available reports suggest that modifying the route of administration may allow for continuation of therapy.

## Conflicts of interest

None disclosed.
